# Identification of the *HMGA2*::*CIBAR1-DT* fusion transcript in two lipomas with chromosomal rearrangements involving chromosomes 8 and 12

**DOI:** 10.3389/fonc.2025.1717031

**Published:** 2026-01-06

**Authors:** Marta Brunetti, Kristin Andersen, Ingvild Lobmaier, Francesca Micci

**Affiliations:** 1 Section for Cancer Cytogenetics, Institute for Cancer Genetics and Informatics, The Norwegian Radium Hospital, Oslo University Hospital, Oslo, Norway; 2 Department of Pathology, The Norwegian Radium Hospital, Oslo University Hospital, Oslo, Norway

**Keywords:** CIBAR1 divergent transcript (CIBAR1-DT), cytogenetics, fluorescence *in situ* hybridization, fusion gene, high mobility group AT-hook 2 (HMGA2) gene

## Abstract

**Background/Aim:**

Lipomas are benign tumors of adipocytic origin. The most frequent chromosome rearrangement in lipomas involves the high-mobility group AT-hook 2 gene (*HMGA2*), which maps to 12q14. We investigated two lipomas showing rearrangements of chromosomal bands 8q22 and 12q14 in their karyotypes, with the aim of identifying the gene products affected by these aberrations.

**Materials and Methods:**

The two lipomas were selected because their karyotype showed an 8;12-rearrangement with the very same breakpoint position. The cases were investigated using RNA sequencing, reverse transcription polymerase chain reaction (RT-PCR), Sanger sequencing techniques, Fluorescence *in situ* hybridization (FISH), and array comparative genomic hybridization (aCGH).

**Results:**

RNA sequencing and RT-PCR of the two lipomas showed the presence of the *HMGA2::CIBAR1-DT* chimera.

**Conclusions:**

The *HMGA2::CIBAR1-DT* fusion, identified here for the first time, is a recurrent transcript in lipomas.

## Introduction

According to the World Health Organization (WHO), lipomas are classified as benign adipocytic tumors composed of mature fat tissue (1). They are the most common mesenchymal neoplasm in adults, more frequently seen in men (2, 3). The most common chromosomal rearrangement in lipomas involves chromosomal band 12q14, targeting the high mobility group A2 (*HMGA2*) gene (1). *HMGA2* has an important role in tumor pathogenesis. In lipomas, the gene is most frequently found truncated; however, its involvement in fusion transcripts with different partners is also well documented (4, 5). In both scenarios, the molecular product is the loss of the 3’ untranslated region (3’ UTR) of *HMGA2*, which normally contains binding sites for microRNAs such as let-7, leading to overexpression of the protein (4, 6). In some cases, gene fusions are formed. The most frequent translocation, t(3;12)(q27~28;q13~15), generates an *HMGA2::LPP* fusion (7), however, other common partners are Phospholipid Phosphatase 3 (*PLPP3*; mapping on1p32.2), Atypical Chemokine Receptor 3 (*ACKR3*; on 2q37), EBF Transcription Factor 1 (*EBF1*; on 5q33), Nuclear Factor I B (*NFIB*; on 9p22), Glutamate Receptor Interacting Protein 1 (*GRIP1*; on 12q14.3), LHFPL Tetraspan Subfamily Member 6 (*LHFP*; on 13q12), SET Binding Protein 1 (*SETPB1*; on 18q12.3), and Gelsolin (*GSN*; on 9q33.2) (5, 8–14). In these fusion transcripts, the DNA-binding AT-hook domains of HMGA2 are retained and fused with the transcriptional regulatory domains of the fusion partner.

Here, we describe a novel, recurrent aberration of chromosome bands 8q22 and 12q14 in lipomas and their molecular product.

## Materials and methods

### Patients

The two lipoma samples were surgically removed at the Radium Hospital, Oslo University Hospital, in the years 2016 and 2021. They were cytogenetically investigated as part of our diagnostic routine. They were located on the flank (case 1) and on the thigh (case 2; **Table 1**). The study was approved by the Regional Ethics Committee (Regional komité for medisinsk forskningsetikk Sør-Øst, Norge). All patients’ information has been de-identified.

**Table 1:** Clinical and karyotypic data for the two lipoma samples investigated.

Sample	Sex/Age	Region	Size (cm)	Karyotype
1	F/58	Flank	7 x 4.7 x 9.5	46,XX, ins(8;12)(q22;q13q14),?t(11;16)(p15;p12)[8]/46,XX[2]
2	F/71	Thigh	13 x 9 x 3	46,XX,t(8;12)(q21;q14)[10]/46,idem,t(5;15)(q13;q15),t(9;20)(q12;p11),del(10)(p13)[cp5]

### G-banding and karyotyping

Samples from the surgical specimens were short-term cultured, stained for G-banding analysis, and cytogenetically characterized as previously described (15, 16). The karyotypic description followed the International System of Cytogenomic Nomenclature (17).

### DNA and RNA extraction

Fresh-frozen material from a representative area of the tumors was used to extract DNA and RNA. DNA was extracted using the Maxwell 16 extractor (Promega, Madison, WI, USA) and purified using a Maxwell 16 Cell DNA Purification kit (Promega) according to the manufacturer’s recommendations. RNA was extracted using a miRNeasy kit (QIAGEN, Hilden, Germany). The concentrations were measured using a QIAxel microfluidic UV/VIS spectrophotometer (QIAGEN) and a Quantus fluorometer (Promega). RNA quality was assessed with an Agilent RNA 6000 Nano total kit on an Agilent 2100 Bioanalyzer (Agilent Technologies, Santa Clara, CA, USA).

### RNA sequencing

Each tumor’s total RNA (200 ng) was sent to the Genomics Core Facility at the Norwegian Radium Hospital, Oslo University Hospital, https://oslo.genomics.no/, for high-throughput paired-end RNA sequencing. The FusionCatcher (18) and DeFuse software package (version 0.6.1) was utilized to identify putative fusion transcripts by analyzing the output generated from RNA-Sequencing (19).

### Reverse transcription polymerase chain reaction

The transcripts identified by RNA sequencing were tested by reverse transcription (RT), polymerase chain reaction (PCR), and Cycle sequencing. In brief, 200 ng total RNA was reverse-transcribed in a 20 μL reaction volume using iScript Advanced cDNA Synthesis Kit for RT-qPCR according to the manufacturer’s instructions (Bio-Rad, Hercules, CA, USA). cDNA was used as a template in subsequent PCR amplification using the primers combination HMGA2-947FW (5’-AGGCAGCAAAAACAAGAGTCCC-3’) and 8q22-intron-SEQ3-94333290-rev3 (5’-GACATTCTGGACCAGGTAGAAGAGA-3’). The quality of cDNA synthesis was assessed by amplification of a cDNA fragment from the ABL proto-oncogene 1, non-receptor tyrosine kinase (*ABL1*) gene using the primer combination ABL1-185F1 (5’-ATGACCCCAACCTTTTCGTTGCA- 3’) and ABL1-325R1 (5’-TAGTTGCTTGGGACCCAGCCTTG-3’). Four μl of the PCR products were stained with GelRed (Biotium, Hayward, CA, USA), analyzed by electrophoresis through a 1.0% agarose gel, and photographed. The remaining PCR products were purified with the MinElute PCR Purification Kit (Qiagen) and sequenced using the BigDye™ Terminator v1.1 Cycle Sequencing Kit according to the company’s recommendations (ThermoFisher Scientific, Waltham, MA, United States). The basic local alignment search tool software (BLAST; https://blast.ncbi.nlm.nih.gov/Blast.cgi) was used for computer analysis of sequence data (20). The BLAT alignment tool and the human genome browser at the University of California, Santa Cruz (UCSC) were also used to map the sequences on the Human GRCh37/hg19 assembly (BLAT; http://genome.ucsc.edu/cgi-bin/hgBlat) (21).

### Fluorescent *in situ* hybridization

FISH analysis was performed on tumor cells from cases 1 and 2 using an *HMGA2* Break-apart Probe MPP16360 (CytoCell, Milton, Cambridge, UK) with the target chromosomal region of 12q14.3, encompassing the *HMGA2* gene. Chromosome preparations were counterstained with 0.2 μg/ml DAPI and overlaid with a 24×50 mm2 coverslip. Fluorescent signals were captured and analyzed using the CytoVision system (Leica Biosystems, Newcastle, UK).

### Array comparative genomic hybridization

Array Comparative Genomic Hybridization (aCGH) was used to examine imbalances in the genome. The CytoSure Consortium Cancer + SNP arrays (Oxford Gene Technology, Oxford, UK) were used according to the manufacturer’s recommendations. The slide (CytoSure Cancer +SNP array, 4 × 180k) was scanned in an Agilent SureScan Dx microarray scanner using Agilent Feature Extraction Software (version 12.2.0.7), and data were analyzed using CytoSure Interpret Software (version 4.11.39, Oxford Gene Technology). The copy number aberrations were identified using the Circular Binary Segmentation (CBS) algorithm. A custom-made aberration filter defining imbalances was added. Copy number aberrations (CNA) were defined as a region with a minimum of five gained/lost probes. The cut-off value for the mean log ratio was set to a minimum of absolute 0.5, and only genomic imbalances larger than 0, 5 Mb were investigated as CNA. Annotations were based on the human reference sequence GRCh37/hg19.

## Results

**Figure 1:**
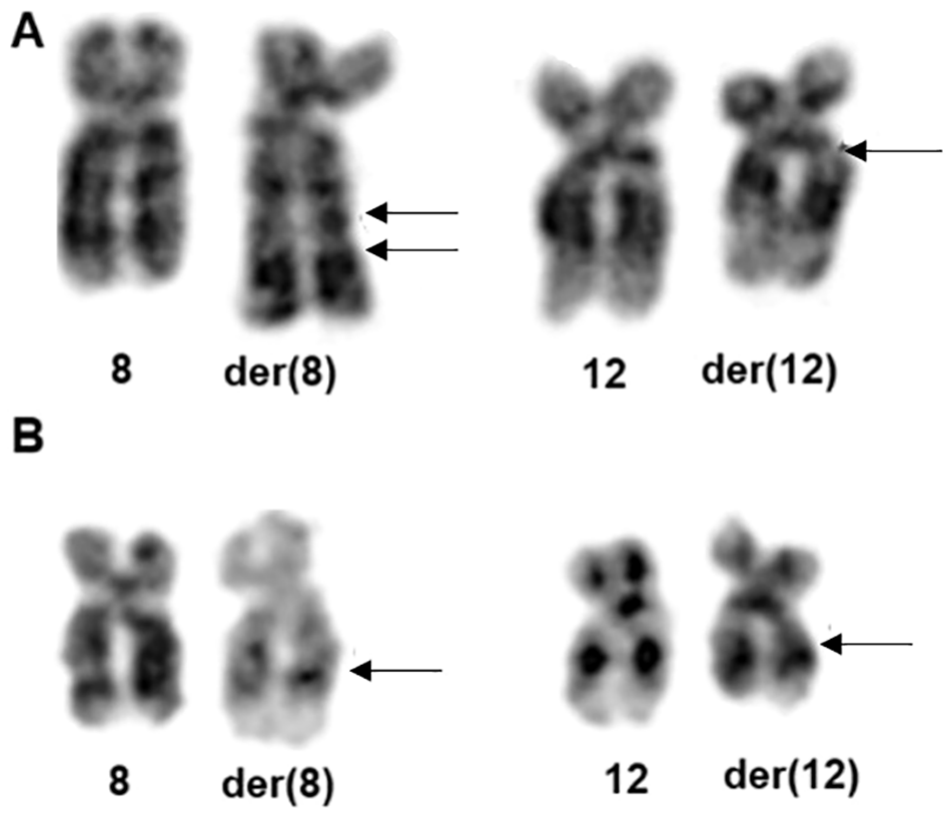
Partial karyograms of cases 1 **(A)** and 2 **(B)** showing normal and derivative (der) chromosomes 8 and 12. Arrows are pointing to the breakpoints position.

The karyotype of both lipomas showed aberrations involving chromosomes 8 and 12 (**Figure 1**), among other rearrangements. In case 1, an insertion of 12q13–q14 into 8q22 was observed, whereas in case 2, a translocation between 8q22 and 12q14 was identified. A complete description of the karyotypes is reported in **Table 1**.

**Figure 2:**
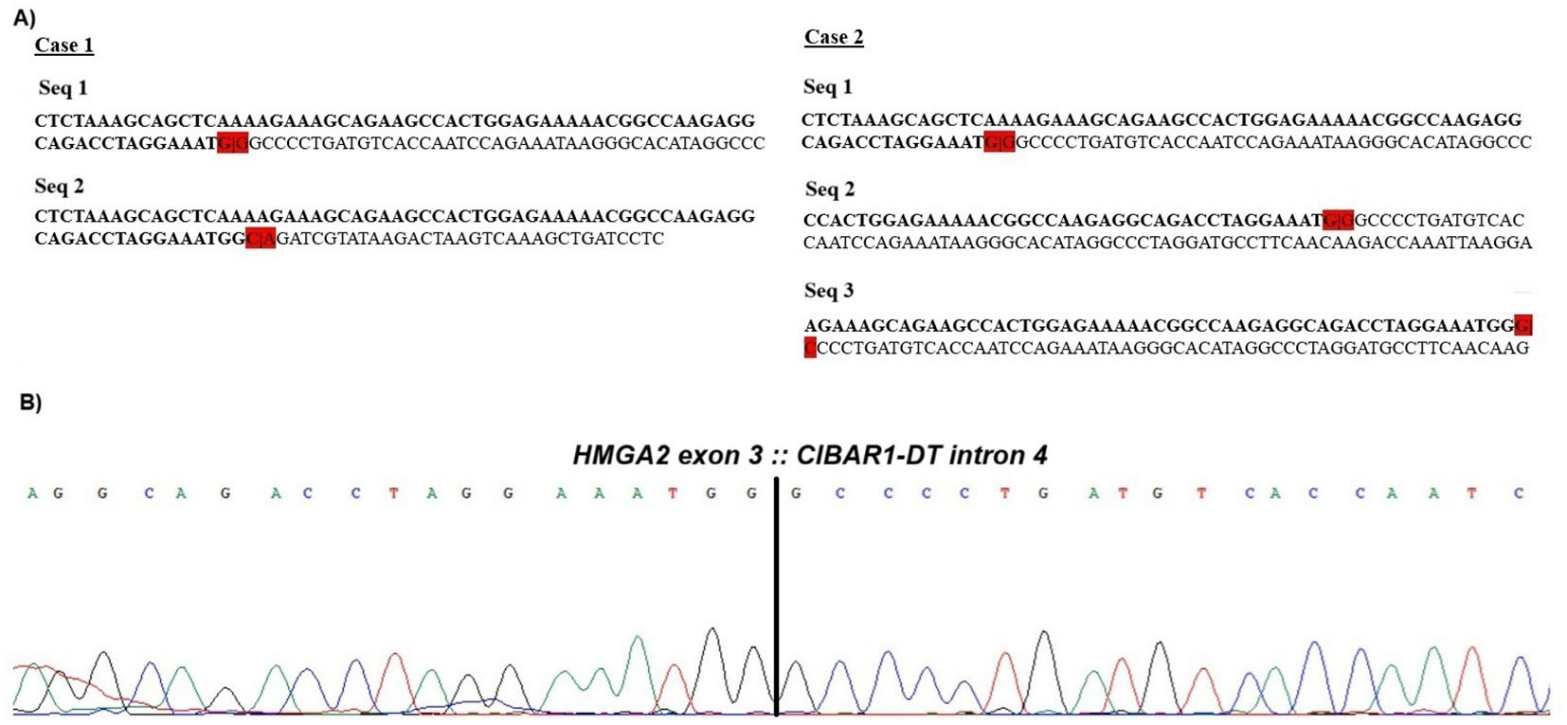
Overview of the sequences found by RNA- and Sanger sequencing. **(A)** The fusion transcripts obtained after analysis using the deFuse software package for cases 1 and 2. In bold: sequence of the high-mobility group AT-hook 2 (*HMGA2*). In red |: the junction of *HMGA2* with a sequence from *CIBAR1-DT*. **(B)** Partial chromatogram of case 1 using the HMGA2-947F and 8q22seq3-rev3 primers. Breakpoint of the fusion transcript is shown between exon 3 in *HMGA2* and intron 4 in *CIBAR1-DT*.

Using the deFuse software on the fastq files of the RNA sequencing data, two *HMGA2::CIBAR1-DT*(previously known as *LINC00535*) chimeric transcripts were found for case 1 and three for case 2. In case 1, the two transcripts involved the exon 3 of the *HMGA2* with introns 4 and 5, respectively, of the *CIBAR1-DT* transcript (ENST00000520096.5). In case 2, the chimeric transcripts involved different breakpoints within the *HMGA2* gene (NM_003483.6), located in intron 2 and exon 3, respectively, and were fused to intron 4 of the *CIBAR1-DT* transcript (**Figure 2**). RT-PCR followed by cycling Sanger sequencing confirmed the presence of the fusion between exon 3 of the *HMGA2* gene and intron 4 of CIBAR1 Divergent Transcript (*CIBAR1-DT*), previously known as Long Intergenic Non-Protein Coding RNA 535 (*LINC00535*)(**Figure 2**), in both cases. The cases were also analyzed with FusionCatcher software, but no fusion transcripts were identified.

**Figure 3:**
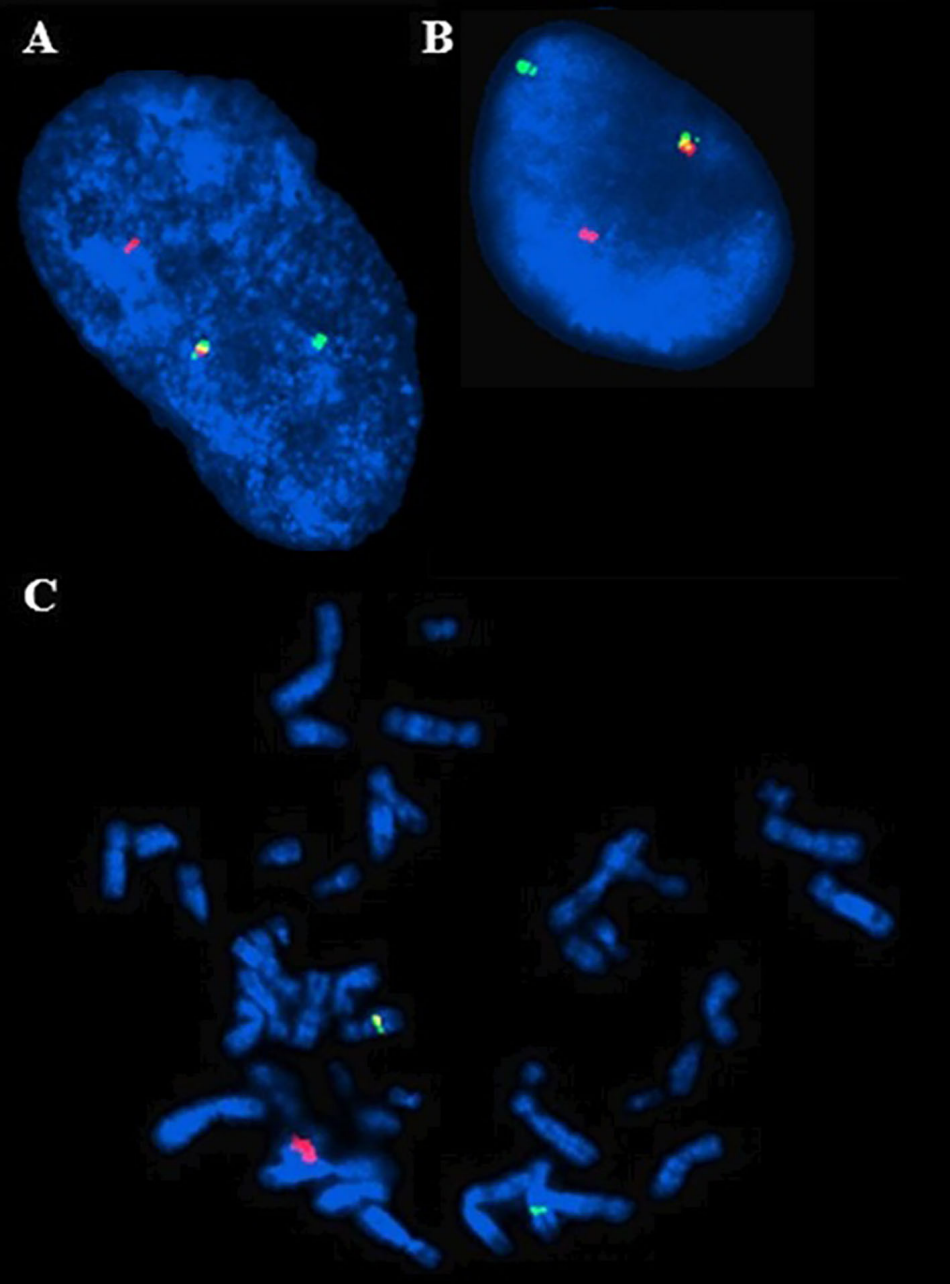
FISH analysis of interphase nuclei from case 1 **(A)** and 2 **(B)** with the *HMGA2* break-apart probe showing the intact *HMGA2* gene (yellow signal) on one of the homologous chromosomes and a disrupted gene in the other (separate red and green signals). FISH analysis on metaphase spread from case 2 **(C)** shows red and green fluorescence signals on derivative chromosome 12 and derivative chromosome 8, respectively, and a yellow signal in the normal chromosome 12.

Examination of metaphase spreads and interphase nuclei, hybridized with the *HMGA2* break-apart probe, showed a split in *HMGA2* (**Figure 3**). The aCGH analysis for cases 1 and 2 revealed no imbalances in the genome when applying the cut-off values routinely used (data not shown).

## Discussion

We present here, for the first time, two lipomas with two 8q22;12q14-rearrangements, leading to the formation of an *HMGA2::CIBAR1-DT* fusion transcript.

The “Mitelman Database Chromosome Aberrations and Gene Fusions in Cancer” database (last updated 10^th^ of July 2025) (22) displays 33 entries of “lipoma” with the involvement of the 12q14/*HMGA2* gene (22). In only 11 entries, the aberration leads to fusion genes involving *HMGA2* with different partners or intergenic sequences. Different types of aberrations were reported; however, no 8q22 involvement has been described. In all types of rearrangement, the sequence of the *HMGA2* gene (exons 1 to 3), coding for the AT-hook domains, is separated from the 3’-untranslated region, which regulates *HMGA2* transcription, resulting in altered expression and leading to tumorigenesis in these tissues (23, 24).

The CIBAR1 Divergent Transcript(*CIBAR1-DT*) is a long non-coding RNA (lncRNA) that maps to chromosome band 8q22.1. It has been reported that lncRNAs are susceptible to form fusion transcripts with gene/mRNA, as it is the case here, as well as with other lncRNAs (25). However, the biological significance of fusions involving lncRNAs in the tumor phenotype remains uncertain (25).

Chromosomal translocations involving the *HMGA2* gene often lead to its truncation. In particular, the coding region of HMGA2 is disrupted, resulting in the loss of critical functional domains required for its architectural role in chromatin remodeling and transcriptional regulation (26). Consequently, the fusion is predicted to cause a loss of function of HMGA2, which may contribute to the pathogenesis of the tumor by impairing its normal gene regulatory functions (27). Since *HMGA2* is important in numerous biological processes, such as cell proliferation and cell cycle progression, the acquisition of sub-microscopic alterations in *HMGA2* can define the pathogenesis of different disease phenotypes (25).

The aCGH analysis did not detect any copy number changes in any of the present cases. This may be due to the log ratio threshold of 0.5 that we chose to score imbalances (28), which may be too stringent to identify low-level mosaic or subclonal copy number changes, as well as lack of good coverage by the platform used. This limitation could be overcome using other type of approach such as chromosomal microarray (CMA) platforms that target high-resolution arrays for the chromosomes in general and the chromosomal sub-band 12q14.3 specifically in the present case, with dense probe coverage of the 3’ UTR region of *HMGA2* (29) and long-read sequencing (30). Both technologies can identify additional cases with cryptic *HMGA2* alterations that remain undetected with conventional FISH or standard aCGH (29, 30). This is the first time rearranged chromosomes 8 and 12 in two lipomas are found to lead to the *HMGA2::CIBAR1-DT* fusion transcript, though with cytogenetically different aberrations, but with the same breakpoints. Cytogenetically abnormal adipocytic tumors with involvement of 8q22 and 12q14, as well as the identification of the *HMGA2::CIBAR1-DT*, can help subclassify these tumors, distinguishing lipomas from malignant adipocytic tumors. The patients with benign lipogenic tumors undergo only a surgical removal of the tumor with no need for further treatment (1).

## Conclusion

In conclusion, aberrations involving the 8q22 and 12q14 chromosomal bands were found to be recurrent in lipomas. The aberrations led to the formation of an *HMGA2::CIBAR1-DT* fusion transcript, which has not been previously identified. The identification of the fusion transcript with involvement of *HMGA2* could be valuable in making a differential diagnosis.

## Data Availability

The original contributions presented in the study are included in the article/supplementary material. Further inquiries can be directed to the corresponding author.
